# Retinal blood flow is increased in type 1 diabetes mellitus patients with advanced stages of retinopathy

**DOI:** 10.1186/s12902-016-0105-y

**Published:** 2016-05-26

**Authors:** Hoang-Ton Nguyen, Eelco van Duinkerken, Frank D. Verbraak, Bettine C. P. Polak, Peter J. Ringens, Michaela Diamant, Annette C. Moll

**Affiliations:** Department of Ophthalmology, VU University Medical Center, De Boelelaan 1117, Amsterdam, 1081 HV The Netherlands; Diabetes Center/Department of Internal Medicine and Department of Medical Psychology/Psychosocial Diabetology Section, VU University Medical Center, Amsterdam, The Netherlands; Department of Psychology, Pontifícia Universidade Católica do Rio de Janeiro (PUC-Rio), Rio de Janeiro, RJ Brazil; Department of Ophthalmology, Academic Medical Center, Amsterdam, The Netherlands; Department of Ophthalmology, VU University Medical Center, Amsterdam, The Netherlands; Department of Ophthalmology, Maastricht University Medical Center, Maastricht, The Netherlands; Diabetes Center/Department of Internal Medicine, VU University Medical Center, Amsterdam, The Netherlands

**Keywords:** Diabetes, Diabetic retinopathy, Hemodynamics, Laser-doppler flowmetry

## Abstract

**Background:**

Diabetic retinopathy (DRP) is a common microvascular complication seen in patients with type 1 diabetes mellitus (T1DM). The effects of T1DM and concomitant (proliferative) DRP on retinal blood flow are currently unclear. Therefore, we measured retinal vascular blood flow in T1DM patients with and without DRP and non-diabetic controls. We further assessed the acute effects of panretinal photocoagulation on retinal microvascular bloodflow in eight patients with diabetes.

**Methods:**

Thirty-three T1DM patients with proliferative DRP, previously treated with panretinal photocoagulation (pDRP), 11 T1DM patients with untreated non-proliferative retinopathy (npDRP) and 32 T1DM patients without DRP (nDRP) were compared with 44 non-diabetic gender-matched controls. Using scanning laser Doppler flowmetry (HRF, Heidelberg) blood flow in the retinal microvasculature was measured temporal and nasal of the optic disc and averaged into one flow value per eye. The right eye was used as a default for further analyses. Eight patients with novel proliferative retinopathy (4 T1DM and 4 with type 2 diabetes) were measured before and several months after photocoagulation. Between-group differences in retinal blood flow were assessed using ANOVA corrected for multiple comparisons (Bonferroni).

**Results:**

Retinal blood flow was higher in the treated pDRP compared with the nDRP group and controls (all *P*_Bonferroni_ < 0.01). Furthermore, there was a positive linear trend for blood flow with lowest blood flow in the control group and highest in the pDRP group (*P*-for-trend < 0.01). In the eight patients with novel proliferative retinopathy, blood flow did not significantly change before and after panretinal photocoagulation (*P* > 0.05). Using regression analysis, no variables were found as predictors of retinal blood flow.

**Conclusions:**

In comparison with controls and nDRP patients, retinal blood flow significantly increased in the pDRP group, which previously underwent photocoagulation treatment, but not in the npDRP patients. These changes may be a consequence of a failing vascular autoregulation in advanced diabetic retinopathy.

## Background

Diabetic retinopathy, a highly prevalent complication of diabetes mellitus, is globally an important cause of blindness in developed as well as developing nations [1, 2]. Chronic hyperglycemia is one of the most important contributing factors [3, 4].

Retinal hemodynamics are thought to be disturbed by progressive diabetic retinopathy. Hitherto, studies assessing retinal blood flow in diabetes patients have shown conflicting results. Several of these studies showed an increased retinal blood flow in diabetes patients with no or minimal retinopathy relative to controls [5, 6], whereas other studies did not note a difference or found decreased blood flow [7, 8]. These differing results could be explained by differences in methodology used, the inclusion of both type 1 and type 2 diabetes patients and sample sizes. The mechanisms of the changing hemodynamic response of the retina in both type 1 and type 2 diabetes are largely unknown. A hypothesis is that chronic hyperglycemia causes loss of pericytes, which play a role in regulating vascular tone, and loss of endothelial cells promotes retinal hemodynamic changes [3]. Alternatively, fluctuations in blood glucose levels known to acutely alter retinal blood flow are also thought to contribute to microvascular damage [9].

To better understand the changes in retinal blood flow in type 1 diabetes we performed a uniform method of retinal flow measurement in patients without retinopathy, with non-proliferative and photocoagulated proliferative retinopathy in comparison with gender-matched non-diabetic controls. To understand the acute effects of laser photocoagulation on retinal blood flow we included 8 patients with diabetes in a substudy and performed the same measurements before and after coagulation.

## Methods

### Study population

For retinal blood flow measurements, 35 patients without DRP, 12 with non-proliferative DRP, 35 with proliferative DRP with T1DM and 45 gender-matched healthy controls were included in this study. This study is a sub-study of a study, which focused on cognitive functioning and brain structure in T1DM, in relation to the presence or absence of proliferative DRP [10]. Participants were recruited from outpatient clinics of the departments of Internal Medicine/Diabetes Center and Ophthalmology, VU University Medical Center, Amsterdam, The Netherlands, and from affiliated hospitals in the Amsterdam area, as well as via advertisements in diabetes specific magazines and a national newspaper. Controls were recruited through participating T1DM patients. To determine the effects of panretinal photocoagulation, we performed a substudy and included 4 type 1 and 4 type 2 diabetes patients newly diagnosed with proliferative retinopathy. Type 2 diabetes patients were included as well, because the aim of this substudy was to investigate changes in blood flow after panretinal photocoagulation, which is applicable to both type 1 and type 2 diabetes patients.

#### Main study: blood flow measurements in type 1 diabetes patients in different stages of diabetic retinopathy

Inclusion criteria for all participants from the first study were: age between 18 and 56 years, proficient in Dutch, right-handedness, and for type 1 diabetes patients a disease duration of at least ten years. Participants were excluded in the presence of visual acuity below 0.3 in either eye; MRI (Magnetic Resonance Imaging)-contraindications, such as ICD (Implantable Cardioverter Defibrillator), pregnancy, non-removable metal implants; history of coma; current or history of cardio- and cerebrovascular disease, and alcohol abuse; current drug use, and use of centrally acting medication including glucocorticoids; or (treatment for) psychiatric comorbidity. Additionally, the following specific ophthalmic exclusion criteria were formulated: macular degeneration or macular edema, cataract, functional monocular vision (identified by the participants’ treating ophthalmologist) and media opacities preventing good quality scans (identified during blood flow measurements). Hypertension was defined as a systolic blood pressure of >140 mmHg, or a diastolic blood pressure of >90 mmHg or use of anti-hypertensive medication. Hypertension, as well as statin use, were additional exclusion criteria for the non-diabetes controls.

To determine the grade of DRP fundus photography (Topcon NW 100, Capelle aan den IJssel, the Netherlands) was performed (EvD) and retinopathy grading was performed independently by 2 ophthalmologists (AM and FV) using the European Diabetes (EURODIAB) classification [11]. For each eye, one photograph centered on the macula and one nasally with the optic disc in the center, was taken (EvD). Only patients with a EURODIAB score of 0 (no retinopathy), 1 (minimal non-proliferative retinopathy) and 5 (treated with panretinal photocoagulation) were included in this study.

#### Substudy: changes in blood flow after panretinal photocoagulation

For measurement before and after panretinal photocoagulation, patients 18 years of age or older and of any diabetes duration with a EURODIAB score of 4 (proliferative retinopathy) were included. For this study, both type 1 diabetes mellitus (T1DM) patients and type 2 diabetes mellitus (T2DM) patients were included. Exclusion criteria for this group were the same as the ophthalmic exclusion criteria mentioned above. Instead of fundus photography, these participants were graded by indirect fundoscopy (TN and AM) or fluorescence angiography (AM), confirming proliferative diabetic retinopathy.

The tenets from the declaration of Helsinki were followed and written informed consent was obtained from all participants. Ethics approval was obtained from the institutional Ethics Review Board.

### Study design

For the main study, during an afternoon visit, blood samples were drawn for routine assessment, including HbA1c and lipid levels and 24-h urine sampling was performed to determine albumin-to-creatinine ratio. During this visit, retinal blood flow was measured and capillary current blood glucose level was determined using finger prick prior to and after blood flow measurements.

Current blood glucose levels were actively kept between 4 and 15 mmol/l. If blood glucose levels were below 4 and above 15 mmol/L, these values were corrected according to a standardized protocol until they reached the required range [10]. Eight patients had a blood glucose level below 4 mmol/l and eight above 15 mmol/l. Thirty minutes after correction of blood glucose levels all participants had levels within the required range and blood flow testing was started.

### Retinal blood flow

Scanning laser Doppler flowmetry is a non-invasive method to measure retinal blood flow [12, 13]. The principle of scanning laser Doppler flowmetry has been described in detail elsewhere [12]. Using the Heidelberg Retinal Flowmeter (HRF) (Heidelberg, Germany), retinal blood flow images were obtained. Flow measurements were made without pupillary dilatation. A scanning window of 10° was used. For measurement of the right eye, subjects were instructed to look into a fixation point with the left eye. The lens was held at a minimum distance of two cm to the cornea of the eye [14].

**Fig. 1:**
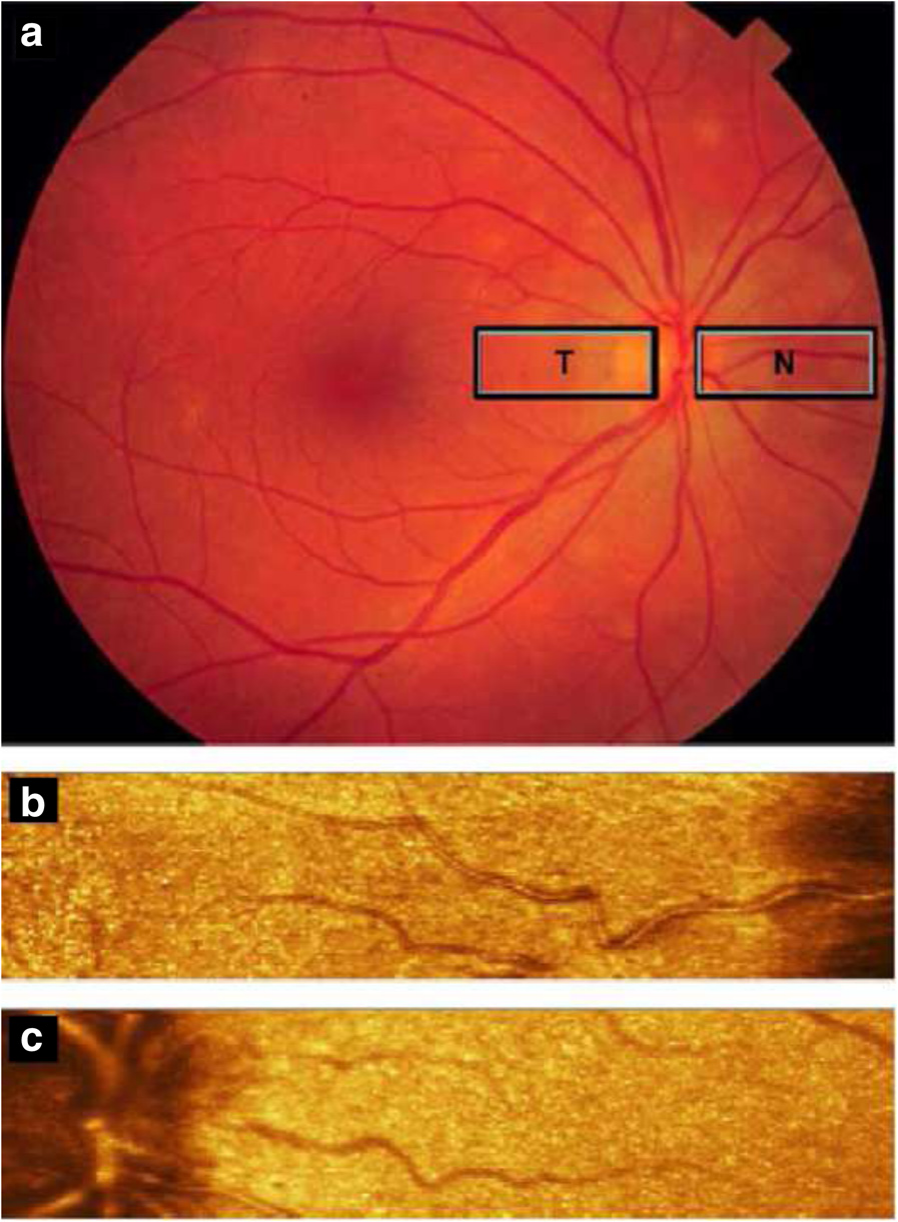
Retinal blood flow images. Image **a** is a fundus photograph of the retina (right eye). The rectangular areas are the temporal (T) and nasal (N) measurement areas. Image **b** and **c** are an example of an image taken with the Heidelberg Retinal Flowmeter of the temporal (B) and nasal areas (C) respectively

Subjects were seated at least ten minutes before starting image acquisition. Retinal blood flow was assessed temporal and nasal to the optic disc (Fig. 1). Three consecutive images were obtained from each area and flow values, obtained with specialized software, were averaged. Larger retinal vessels were used as landmarks to ensure that identical sampling areas were scanned during consecutive measurements. Only subjects with two or more successful measurements for each area were included (mean: 2.65 measurements). Measurements for the substudy before panretinal coagulation were taken on the same day of the scheduled first visit for photocoagulation treatment.

### Image analysis

Offline, the Automated Full Field Perfusion Image Analyser (AFFPIA) version 3.3, which has been validated as reliable analyzing software by Michelson et al. [15], was used to analyze the retinal blood flow images. Using Fourier Transformation, two-dimensional flow maps were obtained. Large vessels and photocoagulation scars were manually excluded, leaving only capillaries to be analyzed. Images were excluded when containing less than 10 % pixels. Flow values of the same scanning area of each subject were then averaged. After analysis, the blood flow value of the measured location was given in arbitrary units (AU). Within each eye the average of the flow values nasally and temporally to the optic disc was taken as single final microvascular flow measurement for each subject.

### Statistical analysis

Statistical analysis was performed using SPSS 15.0 (SPSS, Chicago, IL).

Data were calculated as mean (±SD) for normally distributed or median (with IQR) for non-normally distributed variables. Cohen’s kappa coefficient was used to calculate intra-observer reliability. Group differences for demographical variables were analyzed using an one-way ANOVA with Bonferroni correction for continuous variables and chi-square for categorical variables. Because of non-normal distribution, retinal blood flow values were log-transformed to allow parametric testing. Retinal blood flow values were normally distributed after log-transformation. Student’s *t*-test was used to calculate differences between the left and right eye and between the two measurement areas temporally and nasally to the optic disc, before averaging both values into one value. Linear regression was used to determine confounders. Differences in retinal blood flow were analyzed using ANOVA, with Bonferroni correction for multiple comparisons, and further corrections for confounders. The level of significance was set as *P* = 0.05.

#### Substudy

Student’s *t*-test was used to calculate differences in retinal blood flow before and after panretinal photocoagulation. The level of significance was set as *P* = 0.05.

## Results

### Subjects

Of all subjects included, two patients with proliferative DRP, 1 with non-proliferative DRP, three without DRP and 1 control subject were excluded from further analysis due to insufficient quality of the measurements. Since there were no significant differences between the right and left eye for all groups separately (*P* > 0.05), the right eye was used as default for further analyses, as most data were available for the right eye. If data of the right eye were unavailable, the left eye was used instead. For the final analyses 33 patients with proliferative DRP, 11 with non-proliferative DRP, 32 without DRP and 44 controls (110 right eyes and 10 left eyes) were included. Table 1 displays subjects’ characteristics.

**Table 1:** Demographic and laboratory variables in T1DM patients and controls

	Controls	T1DM no DRP	T1DM non-proliferative DRP	T1DM proliferative DRP (treated)	*P*
N	44	32	11	33	
Age (years)	38 (11.3)	38 (8.7)	36 (5.4)	45 (7.7)	<0.001
Gender (m/f)	18/26	10/22	4/7	15/18	0.687
Systolic blood pressure (mmHg)	124 (12)	131 (17)	121 (11)	136 (19)	<0.01
Diastolic blood pressure (mmHg)	78 (8)	77 (9)	76 (5)	77 (9)	0.941
Diabetes duration (years)	–	22 (8.5)	22 (7.6)	34 (6.6)	<0.001
Hypertension (%)^a^	–	8 (25 %)	2 (18 %)	21 (66 %)	<0.001
Current blood glucose (mmol/l)^b^	–	8.2 (3.2)	8.4 (3.2)	9.7 (5.0)	0. 321
HbA1c (%)	5.3 (0.2)	7.9 (0.9)	7.8 (1.1)	8.1 (1.4)	<0.001
Albumin-to-creatinine ratio^c^	–	0.8 (1.0)	0.5 (0.6)	3.3 (6.7)	0.059
Neuropathy (%)^d^	–	1 (3)	0	15 (47)	<0.01
Nephropathy (%)^e^	–	0	0	6 (19)	<0.01

Data are mean ± standard deviation or absolute number with percentages. *P*-values for overall one-way ANOVA are shown

^a^Hypertension was defined as a systolic blood pressure of 140 mmHg or above, a diastolic blood pressure of 90 mmHg or above or use of anti-hypertensive medication

^b^Current blood glucose level was measured using a finger prick

^c^Albumin-to-creatinine ratio was determined in 24-h urine sampling

^d^Neuropathy was self-reported or obtained from medical records

^e^Nephropathy was based on a 24-h urine sample and defined as an ACR of 2.5 or above for men and 3.5 for women

Groups differed with regard to age, diabetes duration, systolic blood pressure, HbA1c and hypertension (all *P* < 0.01). Post-hoc testing showed that the group with proliferative DRP was significantly older than the group without DRP and controls, had higher systolic blood pressure relative to the patients without DRP and control subjects and had longer disease duration than the group without DRP (all *P* < 0.05). There was no statistically significant difference in mean current blood glucose among the T1DM groups (*P* > 0.05).

### Retinal blood flow

To assess intra-observer reliability, ten images from different subjects were randomly selected to be analyzed with the AFFPIA software by the same observer (HN) six months after initial analysis. The intra-class correlation coefficient (κ) was 0.99 for both the temporal and nasal measurement areas, which is in line with previous literature [16].

**Fig. 2:**
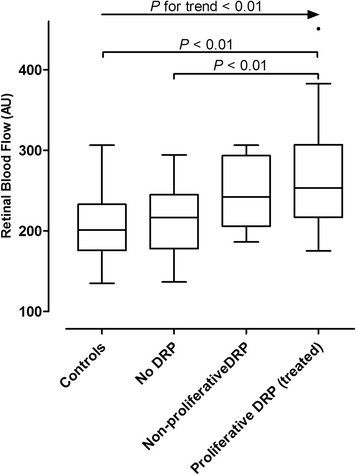
Blood flow values. Box-plot of absolute retinal blood flow values (in Arbitrary Units) showing medians, quartiles, maximum and minimum values. Significant *P*-values for the linear trend and post-hoc tests pairwise comparisons after MANCOVA are shown. Controls (*N* = 44), no retinopathy (*N* = 32), non-proliferative retinopathy (*N* = 11), proliferative retinopathy (*N* = 33)

Figure 2 shows retinal blood flow values (in AU) of all groups. Median flow value (in AU) with corresponding interquartile ranges was 200.9 (±55.0) for controls, 216.8 (±67.2) for nDRP, 229.5 (±87.8) for npDRP and 253.1 (±89.9) for pDRP. Blood flow in the pDRP group was significantly increased compared to the nDRP group (mean absolute difference: 47.4; mean log difference: 0.198; 95 % Confidence Interval (CI): 0.036–0.331; *P* = 0.001) and compared with controls (mean absolute difference 59.5; mean log difference: 0.255; 95 % CI: 0.131–0.378; *P* = 0.000). Between groups, there was a significant positive linear trend for blood flow (*P* < 0.01; Fig. 2) with lowest flow in the control group and increasing with diabetic retinopathy severity. This trend remained significant (*P <* 0.01) after excluding the DRP group from the analysis. Using linear regression, blood glucose levels, HbA1c, diabetes duration, age, gender, hypertension, albumin/creatinine ratio and various other variables were not found to be predictors of retinal blood flow for all groups (*P* > 0.05).

**Fig. 3:**
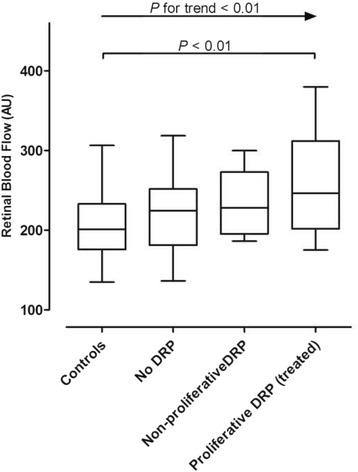
Blood flow values after excluding hypertensive T1DM participants. Box-plot of absolute retinal blood flow values (in Arbitrary Units) showing medians, quartiles, maximum and minimum values. Significant *P*-values for the linear trend and post-hoc tests pairwise comparisons after MANCOVA are shown. Controls (*N* = 44), no retinopathy (*N* = 12), non-proliferative retinopathy (*N* = 9), proliferative retinopathy (*N* = 24)

As hypertension can significantly influence blood flow measurements we have additionally repeated the analysis in patients and controls without hypertension only. Firstly, median flow for T1DM patients with hypertension did not significantly differ from the flow in patients without hypertension (236.7 and 249.7 AU respectively [*P* = 0.170]). After excluding participants with hypertension, an analysis was performed with 12 patients in de pDRP group, 9 patients in the npDRP group and 24 patients in the nDRP group. The median flow for the pDRP group was 246.4 AU, for the npDRP group 228.15 AU and for the nDRP group this was 224.4 AU. As none of the controls were excluded, the median flow in this group did not change (200.9 AU). Despite the apparent loss of sample size and thus statistical power, in the presence of an increased standard deviation this exploratory analysis showed results similar to the whole group analysis. There was an overall effect of group (*P* = 0.001) and a significant linear trend over the groups (*P* < 0.01, Fig. 3). After Bonferroni correction blood flow was higher in the pDRP group compared to controls (*P* = 0.003). There were no significant differences in blood flow between the pDRP group and the other groups.

### Substudy: retinal blood flow before and after panretinal photocoagulation

Eight eyes of 8 participants (4 T1DM patients and 4 T2DM patients) were included for blood flow measurements before and after panretinal photocoagulation (PRP). Table 2 displays subjects’ characteristics.

**Table 2:** Demographic and laboratory variables in T1DM and T2DM patients before and after panretinal laser photocoagulation

Subject no.	Age (years)	Diabetes type	Diabetes duration	Blood pressure (mmHg)	Blood glucose (mmol/L)	IOP (mmHg)	Follow up time (months)
1	63	1	34 years	145/78	6.5	20	2
				143/70	15.7	18	
2	47	1	16 years	127/92	7.2	14	7
				130/96	8.9	15	
3	45	1	30 years	144/85	13.5	13	10
				140/82	12.6	13	
4	24	1	16 years	145/83	5.6	14	12
				122/80	5.6	16	
5	46	2	20 years	138/78	5.9	12	3
				138/78	15.8	14	
6	43	2	6 years	170/95	8.5	18	3
				164/84	6.5	16	
7	84	2	21 years	122/81	6.6	13	3
				130/78	7.5	15	
8	42	2	3 months (from diagnosis)	176/94	6.7	20	7
				150/90	5.6	18	

Data shown for blood pressure, blood glucose and intra-ocular pressure (IOP) represents the measurents before (upper) and after (lower) panretinal photocoagulation

**Fig. 4:**
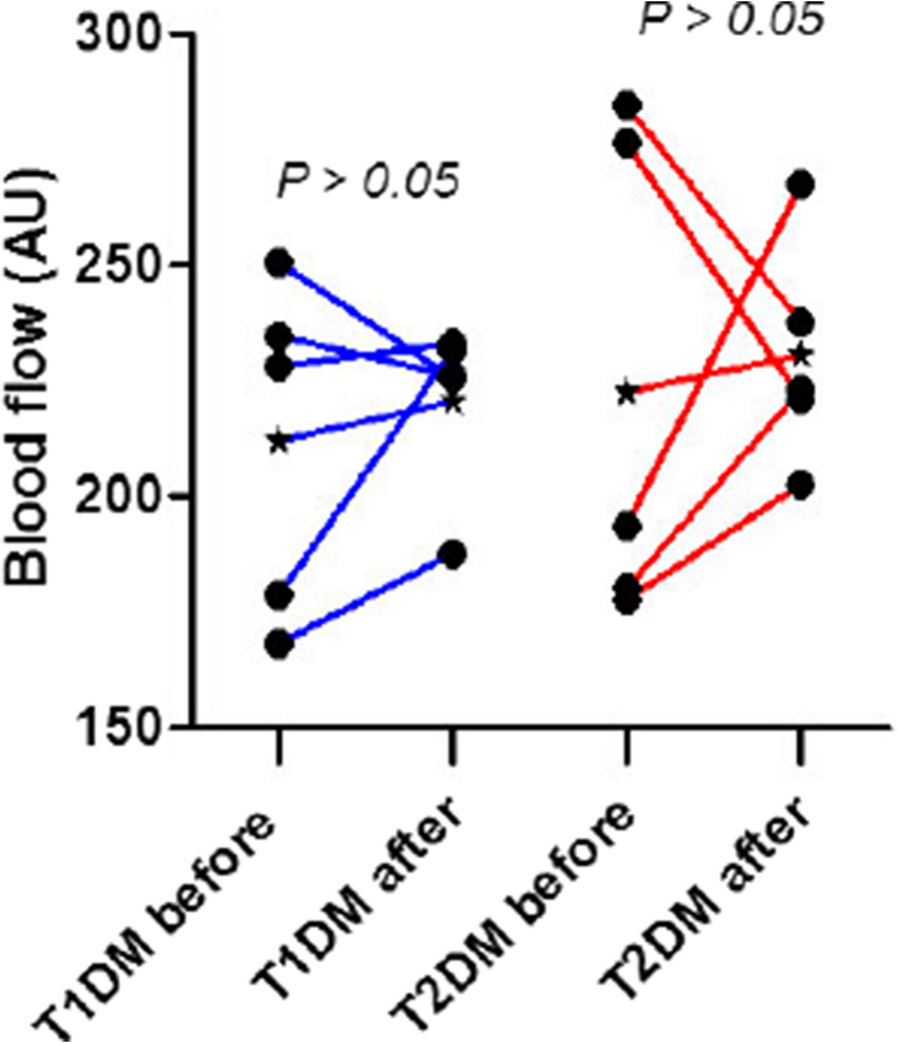
Blood flow before and after panretinal photocoagulation. Individual absolute blood flow values (AU) of 7 right eyes and 1 left eye of patients with type 1 and type 2 DM and proliferative diabetic retinopathy before and after panretinal photocoagulation. The red lines represent the type 1 DM patients and the blue lines represent the type 2 DM patients respectively. The asterisk indicates the mean blood flow values before and after panretinal photocoagulation

Mean age was 48.9 years (range 24–84 years), mean diabetes duration was 19.2 years (3 months to 34 years). Blood glucose in this group ranged from 5.60 mmol/l to 15.8 mmol/l, with a mean of 9.7 mmol/l. Systolic blood pressure ranged from 122 mmHg to 170 mmHg, mean systolic blood pressure was 140 mmHg. Time between completion of panretinal photocoagulation and blood flow measurement ranged from 2 to 12 months. Figure 4 shows retinal blood flow before and after panretinal photocoagulation. Blood flow did not significantly change after panretinal photocoagulation, compared to before panretinal photocoagulation (*P* > 0.05, paired *T*-test). When adding follow-up time as a covariate, change over time remained statistically non-significant (*P* = 0.404). When analyzing the T1DM and T2DM patients separately, we saw similar results in both groups. In the T1DM group, blood flow before panretinal photocoagulation was 211.9 AU, whereas after photocoagulation this increased to 220.7 AU (*P* = 0.28, paired *T*-test). In the T2DM group blood flow before panretinal photocoagulation was 222.4 AU and after photocoagulation increased to 230.6 AU (*P* = 0.303, paired *T*-test).

## Discussion

Our results show increased retinal blood flow in T1DM patients with documented signs of retinopathy. This increased blood flow was most pronounced in patients with treated proliferative retinopathy, although a significant linear trend toward increased blood flow with increasing severity of retinopathy was found in type 1 diabetes patients. This may indicate that retinal blood flow gradually increases in type 1 diabetes patients who develop retinopathy. The increased blood flow was related to the presence of retinopathy itself, but not associated with current blood glucose level, HbA1c, diabetes duration, age, gender, or hypertension. A disturbed retinal blood flow in progressed retinopathy might be expected, since it indicates a damaged microcirculation. Whether retinal blood flow increases in early diabetic retinopathy is unclear, since previous studies focusing on retinal blood flow changes in early stages of retinopathy have reported conflicting results [6–8, 17–20].

Interestingly, in the proliferative DRP group we found a statistically significant higher capillary blood flow as compared to the non-DRP group and controls. This group had previously been treated with panretinal photocoagulation. Studies investigating VEGF levels and blood flow in major retinal vessels suggest a decrease in blood flow after panretinal photocoagulation compared to pre-treatment [21–24]. Blood flow, measured in the larger retinal arteries, was found to decrease after retinal photocoagulation in these studies. To investigate whether panretinal photocoagulation changes retinal blood flow, we measured blood flow in a group of type 1 and type 2 diabetes patients with proliferative diabetic retinopathy before and after panretinal photocoagulation. In partial support of our findings, Cuypers et al. [17], using the same method, reported capillary blood flow to be increased in proliferative retinopathy as measured in the papillo-macular area and unchanged in other parts of the retina [17]. In pDRP two important hemodynamic changes are present. Firstly, this group has a more compromised vascular system with a more decreased autoregulatory function [25]. Secondly, panretinal photocoagulation destroys a significant part of the retinal microcirculation, but larger vessels are left intact. Blood from larger vessels then enters a damaged microcirculation with a diminished number of capillaries. This changed distribution of blood and decreased autoregulation function of the retinal vessels might lead to a higher flow in these capillaries. Capillary drop-out and peripheral areas of non-perfusion are present in proliferative diabetic retinopathy [26]. As we did not find extremely low blood flow values in the pDRP group, it can be assumed that capillary drop-out was modest in the measured areas. The peripheral retina could not be measured using our technique and therefore, and because our measurements were limited to the peripapillary area, we are not able to extrapolate our findings to the entire retina. Hemodynamic changes are thought to parallel diabetic retinopathy progression and possibly even precede visible fundus changes. Studies have found differences in retinal and choroidal blood flow in relation to retinopathy severity [7, 8]. One study found changes in retinal blood flow in diabetic patients without retinopathy [6]. We found a significant positive linear trend for blood flow between the groups. This linear trend indicates that with progressing severity of diabetic retinopathy, retinal blood flow is associated with an increase as well. It may suggest that retinal blood flow is already altered in T1DM without clinically manifest signs of retinopathy. The linear trend persisted after excluding hypertensive patients from the analysis. Furthermore, it is known that elevated blood glucose levels increase retinal blood flow [9]. In this study, we did not find an association between blood glucose levels during retinal blood flow measurement and retinal blood flow. Therefore this could not have confounded our results in de pDRP group.

Both hypertension and vasodilatory medication, such as some antihypertensive medication, could have an effect on blood flow and thus confound our results. Therefore, we repeated our main analysis excluding all patients with hypertension (which also included those using antihypertensive medication). Despite the loss of sample size and statistical power, this analysis showed similar results. Furthermore, blood flow was not significantly different between the groups with and without hypertension and hypertension was not related to blood flow in the regression analysis performed in the whole group. Taken together this suggests that in this group of patients hypertension did not confound our blood flow findings.

Our findings in control subjects differ from the findings of Michelson et al. as they have measured higher blood flow (in Arbitrary Units) [15]. We have measured blood flow with a 790 nm HRF setting whereas Michelson et al. employed a 690 nm HRF setting, which could possibly lead to differences in measurement values. Although overall higher blood flow was found in their study, the relative differences were similar. In their study, blood flow nasally to the optic disc was more increased than temporally to the optic disc, corresponding to our findings.

Although this is a study with well phenotyped T1DM patients with different stages of retinopathy, some limitations have to be mentioned. First, the pDRP group has undergone panretinal laser photocoagulation. Therefore, to determine the effect of panretinal laser photocoagulation, we included several type 1 and type 2 diabetes patients in a substudy. We did not find any significant change in blood flow before and after panretinal photocoagulation, although the substudy was limited by a small sample size and variable time to follow up (2–12 months). This variable follow-up time was due to a number of participants not showing up for the routine check-up after laser photocoagulation and adjusting for this did not change the results. Second, the intraocular pressure (IOP) was not determined in T1DM patients. Substantially elevated IOP would result in lowering of retinal blood flow [27]. Intraocular pressure has been shown to be only marginally elevated in patients with T1DM [28]. Nevertheless, in our study we demonstrated increased blood flow values in the diabetic patients, so the effect of IOP would counteract our results. Control participants were excluded when having hypertension or dyslipidemia. This was chosen as participants included were of a young age (mean age 38 years) with an upper limit of 56 years, and in such young people hypertension and dyslipidemia are often part of the metabolic syndrome or another disorder, which excludes participants from being healthy. Lastly, patients with pDRP are older compared with the other groups. This is a natural phenomenon as it takes 15–25 or more years to reach the proliferative stage of DRP, if it develops at all. Given this age difference, we decided to carefully match in terms of age the control group to the groups without pDRP. To limit the effect of age on the results, it was treated as a confounding factor in all analyses.

Finally it is worth mentioning that measurements with scanning laser Doppler flowmetry must be interpreted with caution. There is ongoing debate on what exactly is measured with this technique. Using experimental models, studies have shown that scanning laser Doppler flowmetry measures blood flow in the larger and smaller elements of the retinal microvasculature [29, 30]. It would have been interesting to also measure the large vessels as well as the choroidal circulation to capture a more complete picture of retinal blood flow. Unfortunately, due to technical limitations of the Heidelberg Retinal Flowmeter and using the AFFPIA software, it is not capable of capturing flow in these locations [31, 32].

## Conclusion

In conclusion, an increased retinal blood flow was observed in T1DM patients with treated and untreated proliferative retinopathy and blood flow seems to increase in increasing severity of retinopathy. We found no difference in retinal blood flow before and after panretinal laser photocoagulation for proliferative diabetic retinopathy. How these hemodynamic changes develop over time and what the underlying mechanisms are, remains unclear. Future prospective research is needed to elucidate the changes in blood flow over time which accompany a failing retinal vasculature in T1DM.

## Abbreviations

AFFPIA: automatic full field perfusion analyser
DRP: diabetic retinopathy
HRF: Heidelberg retinal flowmeter
nDRP: no diabetic retinopathy
npDRP: non-proliferative diabetic retinopathy
pDRP: proliferative diabetic retinopathy
T1DM: type 1 diabetes mellitus
